# Oral myiasis complicating endotracheal intubation in a diabetic patient: a case report

**DOI:** 10.3389/fendo.2026.1820522

**Published:** 2026-05-15

**Authors:** Qimei Xiao, Yawen Zhao, Junyi Du, Yameng Wang, Qi Tang, Zhangrong Liang

**Affiliations:** 1 The Eighth Clinical Medical College, Guangzhou University of Chinese Medicine, Foshan, China; 2 Department of Emergency, Foshan Hospital of Traditional Chinese Medicine, Foshan, China

**Keywords:** diabetes mellitus, nosocomial infection, oral care, oral myiasis, tracheal intubation

## Abstract

Myiasis is a rare parasitic disease caused by infestation of dipterous fly larvae into living human or animal tissues. Although nosocomial myiasis has been occasionally reported in critically ill patients, cases of oral myiasis in patients with diabetic ketoacidosis (DKA) coma undergoing tracheal intubation remain rarely reported in South China. Herein, we report a clinical case of a 77-year-old male with type 2 diabetes mellitus who developed oral myiasis after emergency tracheal intubation for DKA coma. Comprehensive therapeutic strategies were implemented, including manual removal of larvae, oral debridement and irrigation, anti-infective therapy, and intensive glycemic control. To the best of our knowledge, this study is the first to focus on the clinical characteristics and high-risk predisposing factors of oral myiasis in intubated patients with DKA complicated by multiple organ dysfunction and septic shock in the hot and humid climate of South China. Combined with a literature review, we clarify key points of oral care interventions for such critically ill patients. This case supplements clinical data on this rare complication in South China and provides novel clinical references for the prevention, early identification, diagnosis, and treatment of oral myiasis in critically ill diabetic patients undergoing tracheal intubation in the emergency intensive care unit (EICU). Our findings emphasize that individualized oral care should be strengthened for high-risk inpatients who are bedridden, undergo invasive procedures, and present with severe metabolic disorders to prevent such rare nosocomial infections.

## Introduction

Myiasis is a parasitic disease caused by dipteran fly larvae feeding on necrotic or living host tissues (1). It can be classified based on entomological characteristics or clinical manifestations. Entomologically, it is categorized into obligate parasitism, facultative parasitism, and accidental parasitism. Clinically, it is classified according to the site of infestation, including cutaneous, subcutaneous, auricular, ophthalmic, intestinal, and genitourinary myiasis (2). Among these, oral myiasis is insidious and rare, mainly induced by disruption of the oral microenvironment and impaired local defensive functions.

Alterations in the local oral environment are the core prerequisite and critical determinant for the development of oral myiasis. Poor oral hygiene, periodontal lesions, and mucosal injury directly provide a suitable niche for fly oviposition and larval growth. For instance, deep and extensive dental caries and gingivitis lead to massive accumulation of oral secretions, which not only serve as distinct oviposition cues for flies but also supply nutrients for larval development (3). Furthermore, accumulation of necrotic tissues and food residues further optimizes the larval living environment and accelerates their proliferation.

In addition, diabetes mellitus impairs the oral mucosal barrier and reduces neurosensory function (4–6), preventing patients from timely detecting fly oviposition and larval infestation and mounting effective defensive responses, thereby further elevating infection risk. Inadequate oral hygiene management results in persistent accumulation of food residues and secretions, continuously disrupting the integrity of the oral mucosa and forming a vicious cycle that ultimately leads to oral myiasis.

This study reports a case of oral myiasis following tracheal intubation in a patient with type 2 diabetes mellitus in South China. Combined with the clinical management process, we focus on analyzing the mechanistic association between tracheal intubation and oral myiasis and exploring the clinical importance of standardized oral care, aiming to provide a reference for the clinical prevention, early identification, and standardized management of this rare complication.

## Case presentation

On June 9, 2025, a 77-year-old male patient who had been living in a nursing home for a long time was rescued by an ambulance due to “unconsciousness for 1 hour”. At the scene, the patient was in a comatose state, with a body temperature of 38.7 °C, blood pressure of 79/60 mmHg, heart rate of 123 beats per minute, SpO_2_ of 79%, and GCS score of 3. Endotracheal intubation was immediately performed, and the patient was connected to a ventilator for assisted ventilation. Emergency fingertip blood glucose was “Hi”, blood ketone was 2.4 mmol/L, and arterial blood gas analysis showed pH 7.218 and lactate 6.92 mmol/L. Symptomatic treatments such as fluid replacement, hypoglycemic and ketone-lowering therapy, and vasopressor support were given. Considering the critical condition of the patient, the emergency physician admitted him to the Emergency Intensive Care Unit (EICU) with the diagnosis of “coma of unknown cause: coma due to type 2 diabetic ketoacidosis?”.

The patient had long resided in a general nursing home without one-to-one care or professional medical supervision, and received only basic daily care with no regular nursing interventions or standardized management of chronic diseases. He had a history of type 2 diabetes mellitus, sequelae of cerebral infarction, and hypertension for many years. He had not taken any medications regularly, including hypoglycemic agents or antihypertensive drugs. He was bedridden for a long time with limited family care involvement. (Comment 1 from Reviewer 1: The patient’s living conditions in the nursing home and past medical history were supplemented.) After admission to the EICU, his SOFA score was 13, and APACHE II score was 50. Our medical team provided symptomatic treatments such as fluid replacement and ketone-lowering therapy, insulin hypoglycemic therapy, maintenance of electrolyte balance, anti-infection therapy with piperacillin-tazobactam, and high-dose norepinephrine for vasopressor support (1.42 μg/kg/min). From June 10 to 11, the patient’s condition progressed rapidly, with repeated chills and high fever, especially in the early morning, with a peak temperature of 40.4 °C. Infection markers increased progressively (procalcitonin increased from 0.85 ng/mL to 10.36 ng/mL), and troponin TNI increased from 0.10 ng/mL to 3.826 ng/mL, accompanied by multiple organ dysfunction and hemodynamic instability. Considering complicated septic shock, NGS testing and CRRT treatment were recommended, but the family refused. The anti-infection regimen was upgraded to imipenem-cilastatin combined with omadacycline.

**Figure 1:**
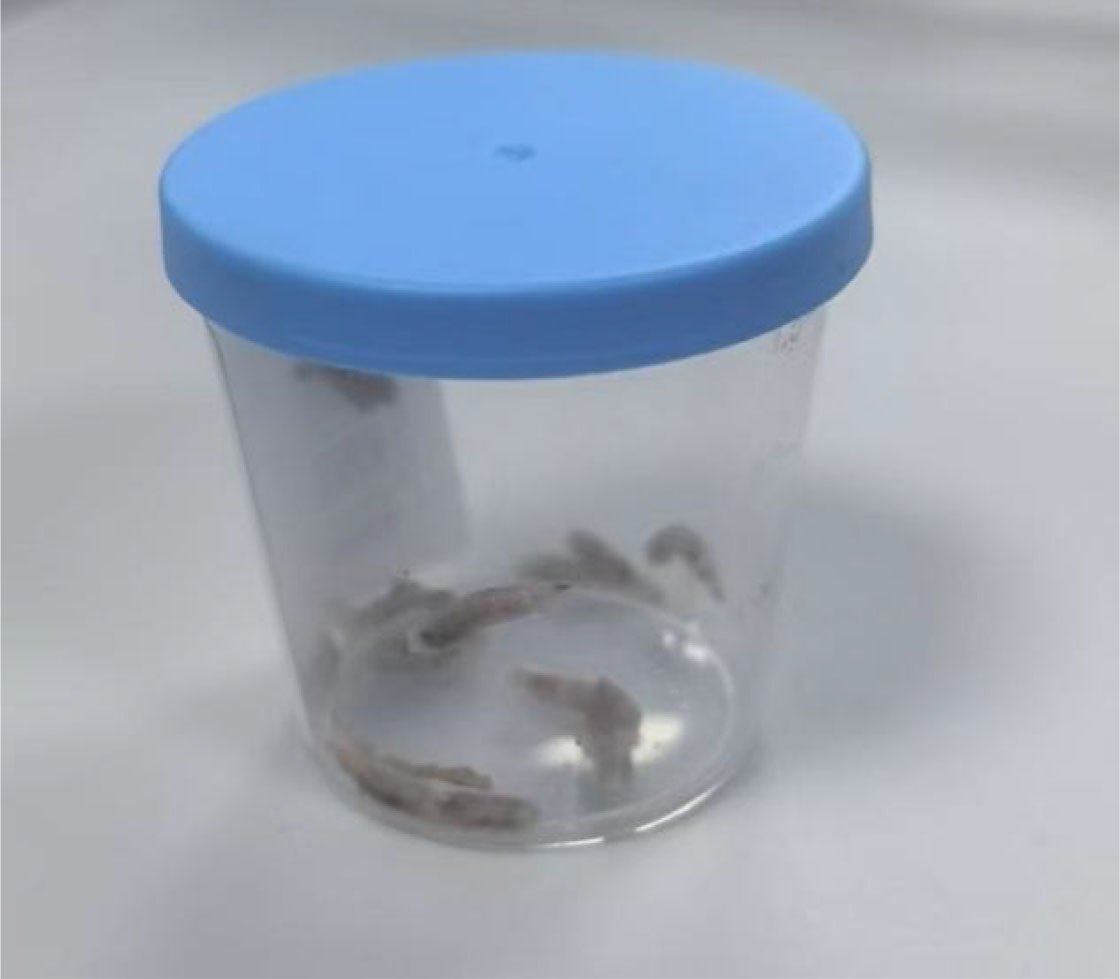
Maggots removed from the oral cavity.

**Figure 2:**
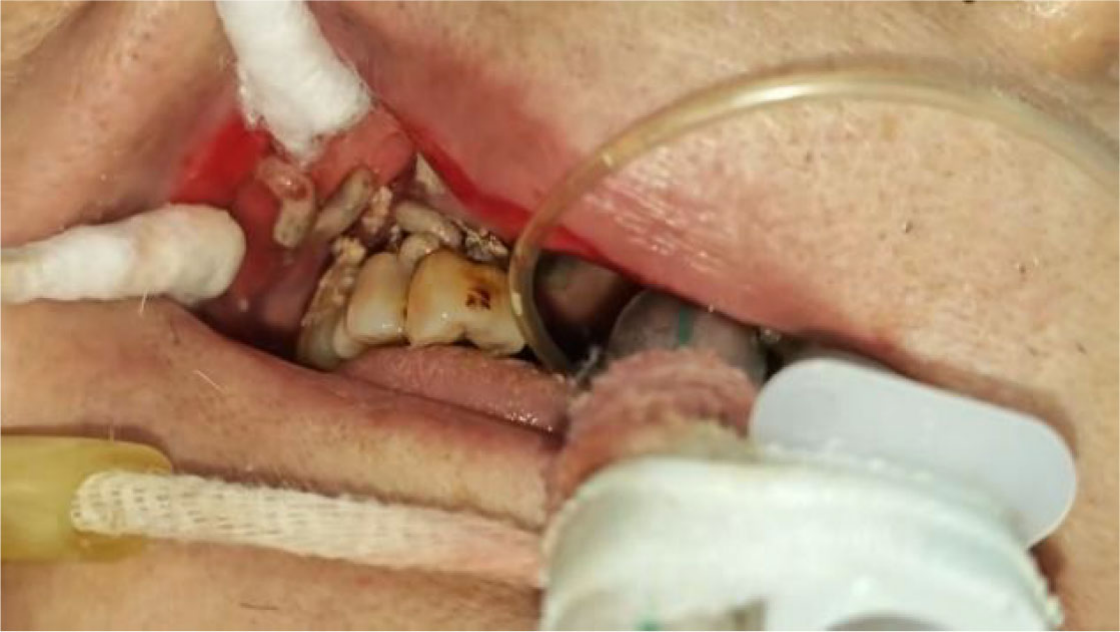
Maggots in the oral cavity.

**Figure 3:**
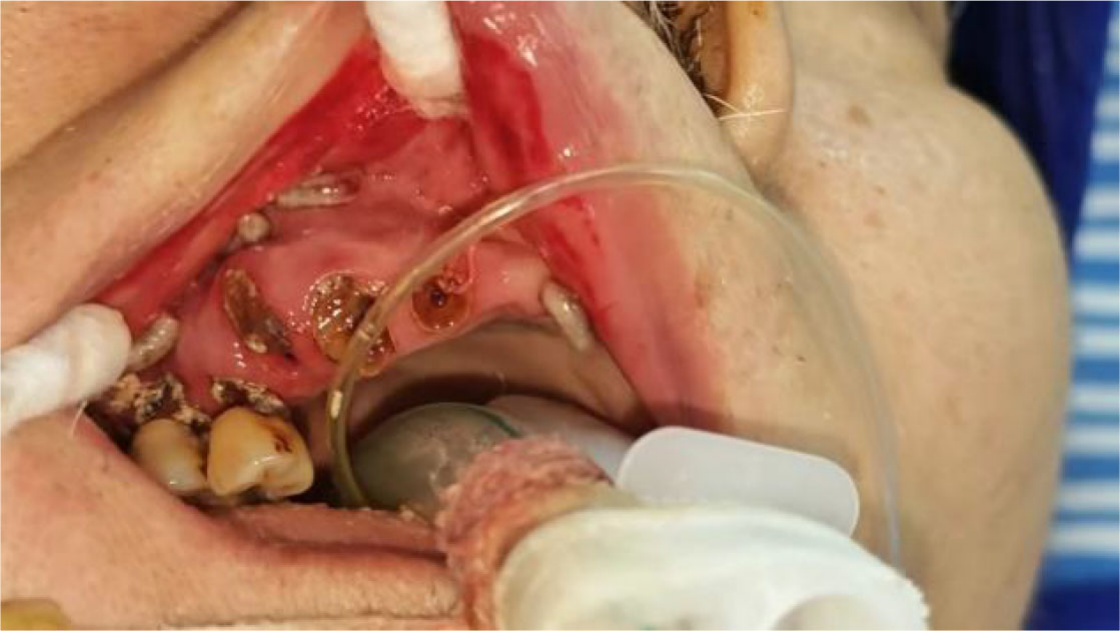
Maggots in the oral cavity.

**Figure 4:**
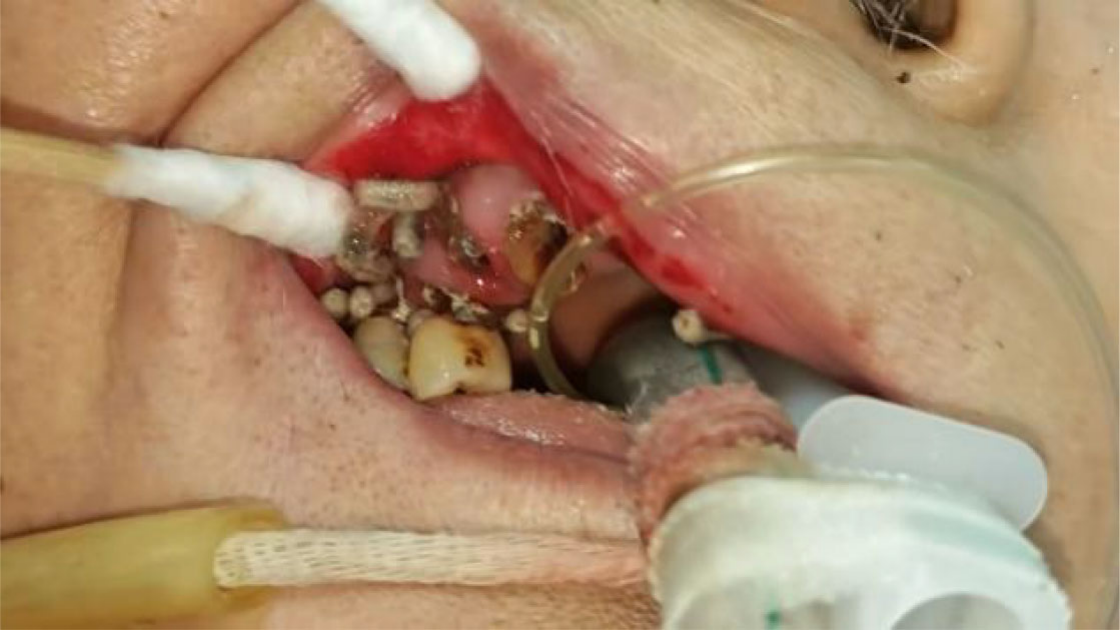
Maggots in the oral cavity.

**Figure 5:**
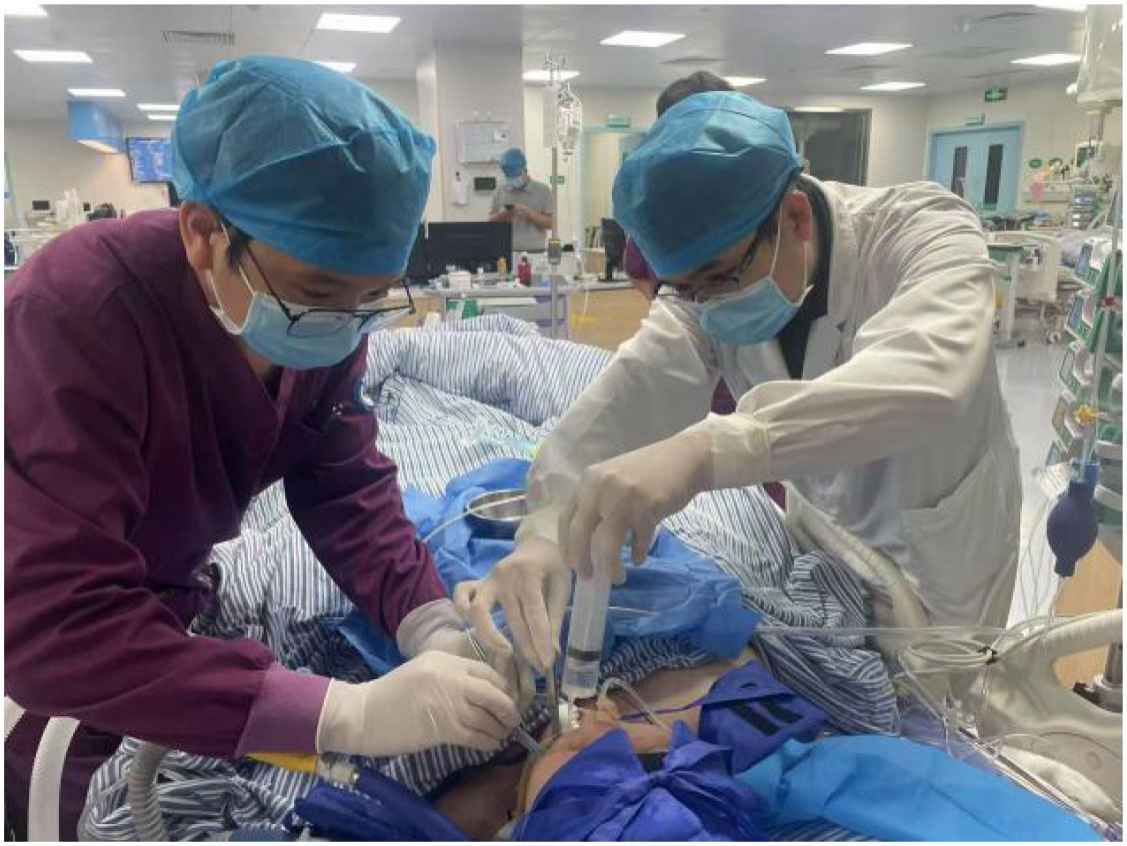
The doctor irrigated the oral cavity with hydrogen peroxide solution.

On June 12, during routine oral care, the nurse found a large number of white wriggling worms in the patient’s mouth, crawling between the lips and teeth (**Figures 1**–**4**). Maggots were suspected, and a consultation with the department of stomatology was requested. The examination showed significant gingival hyperemia and swelling, residual roots of teeth 12, 13, and 14; deep caries on the mesial, buccal cervical, and distal surfaces of teeth 15, 16, and 17, reaching the deep dentin, with soft and moist carious tissue. The gingiva around teeth 15, 16, and 17 was significantly red and swollen, with rounded gingival margins, easy bleeding on probing, edematous gingival papillae, and obvious horizontal resorption of the alveolar crest, reaching the middle 1/3 of the root. Diffuse hyperemia and redness were observed on the inner mucosa of the upper and lower lips and the corner of the mouth. Approximately 20–30 maggots were removed from the mouth with tweezers, followed by local oral irrigation with alternating large amounts of hydrogen peroxide solution, oral and maxillofacial soft tissue debridement, normal saline, and Aneriodine disinfectant (**Figure 5**). as well as oral and maxillofacial soft tissue debridement. Multidisciplinary consultations involving otolaryngology, gastroenterology, infectious diseases, respiratory medicine, and clinical pharmacy were conducted. Sputum culture grew Proteus mirabilis, and blood culture grew Staphylococcus hominis. Tests for Plasmodium spp., Paragonimus spp., Schistosoma spp., Clonorchis sinensis, Echinococcus spp., Taenia solium cysticerci, Spirometra mansoni, and Angiostrongylus cantonensis were all negative. The infection was highly suspected to originate from the oral cavity, with hematogenous or central nervous system infection not excluded. Cranial computed tomography (CT), NGS, and larval morphological identification were recommended but refused by the family. The patient died on the afternoon of June 12, 2025.

## Discussion

Diabetic patients are inherently susceptible to oral myiasis. Persistent hyperglycemia suppresses the function of immune cells such as neutrophils and lymphocytes (7); severe ketoacidosis further inhibits immune cell activity and damages the oral mucosal barrier; electrolyte disturbances impede mucosal repair. Septic shock is characterized by coexisting systemic inflammatory response syndrome and immune paralysis (8), rendering the host incapable of clearing myiasis larvae and exacerbating infection dissemination. Peripheral neuropathy secondary to long-term hyperglycemia causes loss of oral mucosal sensation, eliminating physical defense mechanisms and allowing persistent progression of infection (6).

First, the patient had severe periodontal lesions, deep caries, and residual roots, which constituted the pathological foundation for myiasis infestation. Uncontrolled diabetes exacerbates periodontitis, leading to gingival erosion and mucosal barrier disruption, providing entry sites for fly oviposition. Putrid odors from deep caries and residual roots promote fly attraction and oviposition, while oral debris, secretions, and necrotic tissues supply sufficient nutrients for larval growth. Coma and mouth breathing increase oral humidity, and diabetic neuropathy causes mucosal sensory loss, collectively facilitating the occurrence and progression of oral myiasis.

Secondly, endotracheal intubation performed in this patient may serve as one of the core risk factors contributing to the development of infection. Emergency tracheal intubation for coma resulted in prolonged mouth breathing and disruption of the oral physiological barrier. The patient had hemiplegia due to previous cerebral infarction, was bedridden in a nursing home, and fully dependent on care, with no ability to perform autonomous oral cleaning. Diabetic peripheral neuropathy further caused oral mucosal sensory loss. The combination of these factors created a favorable local microenvironment for fly oviposition and larval propagation. We hypothesize that the patient may have been infested pre-admission, and tracheal intubation exacerbated oral secretion stasis and mucosal exposure, accelerating larval infestation and proliferation. Furthermore, the patient’s extremely critical condition upon EICU admission led clinicians to prioritize life-saving interventions such as hemodynamic stabilization, glycemic control, ketone reduction, and anti-infection, resulting in temporary oversight of oral cleaning and examination for intubated patients, which further increased infection risk and placed the patient in an ultra-high-risk category for oral myiasis.

It should be clarified that the patient’s ultimate death was primarily attributed to multiple organ failure and refractory septic shock induced by diabetic ketoacidosis. Oral myiasis acted as a critical secondary complication that may aggravate systemic infection and inflammatory responses. However, without autopsy results and definitive etiological evidence, its role as the direct cause of death cannot be verified.

This case has several notable limitations. Due to the family’s refusal of further examinations, larval morphological observation and species identification were not completed, leaving the specific pathogenic fly species unidentified. In addition, cranial and chest CT examinations were not performed to exclude intracranial and pulmonary larval infestation. Accordingly, the origin, invasion route, dissemination scope and onset time of the infection remain unclear. These deficiencies limit the exploration of etiological characteristics and transmission mechanisms, prevent targeted clinical intervention and in-depth etiological analysis, and weaken the reliability of diagnostic evidence and the robustness of relevant conclusions. Such deficiencies highlight an important area for improvement in future clinical practice. All above limitations must be fully considered when evaluating the clinical value of this case.

Similar cases of myiasis associated with prolonged bedridden status, inadequate care in nursing facilities, and complications following endotracheal intubation or tracheostomy have been reported worldwide. The core clinical characteristics of these cases are summarized below (**Tables 1**, **2**).

**Table 1:** Summary of clinical characteristics of reported oral myiasis cases in the literature.

Author/year	Age/gender	Comorbidities	Infection site
Ribeiro M C et al./2012 (9)	97/M	Intestinal disease, skin cancer, heart disease, long-term bedridden, cachexia	Oral soft tissues, paranasal sinuses
Rao G S et al./2009 (10)	58/M	Type 2 diabetes mellitus, hypertension, post-mucormycosis surgery, post-tooth extraction	Oral palate, extraction socket, maxillary sinus
Ünalan A T et al./2022 (11)	69/M	Granulomatosis with polyangiitis, myocardial infarction, renal failure, endotracheal intubation, cognitive impairment	Oral cavity
Espinoza-Gómez F et al./2023 (12)	37–83, 3 M & 2 F	Hypertension, cerebral infarction, diabetes mellitus, liver failure, skin cancer, etc.; 3 cases with invasive mechanical ventilation	Oral cavity/nasopharynx/around endotracheal tube, foot ulcer, subcutaneous tunnel
Jang M et al./2013 (13)	37/M	Becker muscular dystrophy, 30-year bedridden, endotracheal intubation, bronchiectasis	Oral cavity
Mowlavi G et al./2011 (14)	63/M	Lung cancer, apnea, deep coma, post-tracheotomy + mechanical ventilation	Tracheotomy site
Taş C Z et al./2019 (15)	63/M	Intracerebral hemorrhage, respiratory failure, hypertension, multiple organ failure, mechanical ventilation	Gingival region
Albarrak Y M et al./2025 (16)	70/F	Type 2 diabetes mellitus, hypertension, dyslipidemia, hypothyroidism, post-stroke coma, post-tracheostomy	Tracheostomy site

**Table 2:** Summary of clinical characteristics of reported oral myiasis cases in the literature.

Author/year	Number & species of larvae	Treatment	Outcome
Ribeiro M C et al./2012 (9)	110; Cochliomyia hominivorax (Calliphoridae)	Mechanical maggot removal + tooth extraction + debridement and suture, ICU symptomatic treatment	Death due to systemic complications 2 days postoperatively
Rao G S et al./2009 (10)	8–10; Musca domestica (Muscidae)	Mechanical removal + irrigation + antibiotics + maggot repellent, subsequent hemimaxillectomy	Well-healed 6 months postoperatively
Ünalan A T et al./2022 (11)	Multiple; Phormia regina (Calliphoridae), 2nd and 3rd instar larvae	Mechanical removal + normal saline irrigation, comorbidity treatment	Infection controlled without recurrence
Espinoza-Gómez F et al./2023 (12)	Approximately 10 per patient; Cochliomyia macellaria (Calliphoridae), 2nd and 3rd instar larvae	Mechanical removal + debridement + antibiotics + ivermectin	2 deaths from septic shock, 3 cured
Jang M et al./2013 (13)	43; Lucilia sericata (Calliphoridae), 3rd instar larvae	Oral suction for larval removal, comorbidity treatment	Cured and discharged
Mowlavi G et al./2011 (14)	Approximately 100; Lucilia sericata (Calliphoridae), 2nd instar larvae	Mechanical removal + wound irrigation + insect repellent	Fatal nosocomial myiasis
Taş C Z et al./2019 (15)	5; Calliphoridae (Diptera), 3rd instar larvae	Mechanical larval removal, symptomatic treatment for comorbidities	Death 6 days after admission
Albarrak Y M et al./2025 (16)	38; Unidentified species	Mechanical removal + iodine solution irrigation + debridement + anti-infection	Cured without recurrence

These cases commonly involved middle-aged, elderly, or young patients with severe comorbidities, characterized by immunosuppression, prolonged bedridden status, poor self-hygiene, and partially invasive interventions; infections occurred in secretion-prone exposed sites (oral cavity, tracheostomy stoma) due to insufficient care and hygiene. The present case shared the same high-risk features and core management (debridement, irrigation, antibiotics), but differed by complicated with diabetic ketoacidosis, septic shock, and multiple organ dysfunction, representing a more critical condition with heavier risk burden, faster progression, severe oral caries and residual roots, and a poorer prognosis.

Most existing cases involve 1–2 risk factors without severe systemic metabolic disorders or multiple organ dysfunction. This case fills the clinical research gap of oral myiasis in critically ill patients with complex high-risk factors and critical illness. It also breaks through the limitation of previous studies focusing only on the local oral effects of hyperglycemia, revealing that DKA-induced acidosis disrupts the oral mucosal barrier and septic shock causes systemic immune failure, which together create a susceptible microenvironment for oral myiasis in diabetic patients, expanding the mechanistic link between diabetes and this disease and providing new directions for oral infection prevention and control in critically ill diabetic patients. The core value lies in the first systematic elaboration of the synergistic pathogenic mechanism of “systemic immune failure–local oral pathological destruction–EICU oral care oversight” in critically ill diabetic patients, supplementing clinical characteristic data of this population, clarifying the specific links of local oral pathology triggering myiasis infestation, and providing new evidence-based references for precise prevention and early identification of this disease.

## Conclusions and recommendations

Myiasis is widely recognized not only as a parasitic disease but also as an important clinical and forensic marker of neglect, poor hygiene, and suboptimal care in vulnerable individuals, including elderly, bedridden, comatose, and critically ill patients. In the present case, the patient was elderly, comatose, bedridden, with multiple comorbidities including diabetes mellitus, and completely dependent on care. The occurrence of oral myiasis strongly suggests insufficient oral hygiene, inadequate environmental protection, and potential deficiencies in daily care. As emphasized in previous forensic studies, myiasis in immobile or unconscious patients should raise high suspicion of neglect and requires careful evaluation of care quality. Bugelli et al. highlighted the medicolegal significance of myiasis as a reliable indicator of neglect and poor care in vulnerable populations, particularly in settings such as nursing homes or long-term care facilities (17). In this case, the combination of advanced age, complete dependency, lack of effective oral care, and family refusal of further examinations eventually led to a fatal outcome, which further supports the view that oral myiasis can serve as a critical warning sign of neglect and substandard care.

In critical care medicine, oral care in critical care is not merely superficial cleaning. For critically ill EICU patients, oral care is a cornerstone of nosocomial infection prevention and control. Inadequate oral cleaning that fails to eliminate debris and secretions sustains a putrid microenvironment; incomplete oral examination that misses early intervention for severe periodontitis and deep caries—oral care serves as the first line of defense against severe complications such as oral myiasis and ventilator-associated pneumonia (VAP). Oversight of oral care essentially reflects insufficient awareness of infection risk in ultra-high-risk EICU patients. This case strongly suggests that oral care should be integrated into the core clinical management workflow of critically ill EICU patients, with heightened emphasis on its pivotal role in infection control.

## Data Availability

The original contributions presented in the study are included in the article/supplementary material. Further inquiries can be directed to the corresponding author.
